# Is the Nintendo Wii Fit really acceptable to older people?: a discrete choice experiment

**DOI:** 10.1186/1471-2318-11-64

**Published:** 2011-10-20

**Authors:** Kate Laver, Julie Ratcliffe, Stacey George, Leonie Burgess, Maria Crotty

**Affiliations:** 1 Department of Rehabilitation and Aged Care, Flinders University, Adelaide, South Australia, Australia; 2 Flinders Clinical Effectiveness, Flinders University, Adelaide, South Australia, Australia; 3 Department of Mathematical Sciences, University of Technology, Sydney, New South Wales, Australia

## Abstract

**Background:**

Interactive video games such as the Nintendo Wii Fit are increasingly used as a therapeutic tool in health and aged care settings however, their acceptability to older people is unclear. The aim of this study was to determine the acceptability of the Nintendo Wii Fit as a therapy tool for hospitalised older people using a discrete choice experiment (DCE) before and after exposure to the intervention.

**Methods:**

A DCE was administered to 21 participants in an interview style format prior to, and following several sessions of using the Wii Fit in physiotherapy. The physiotherapist prescribed the Wii Fit activities, supervised and supported the patient during the therapy sessions. Attributes included in the DCE were: mode of therapy (traditional or using the Wii Fit), amount of therapy, cost of therapy program and percentage of recovery made. Data was analysed using conditional (fixed-effects) logistic regression.

**Results:**

Prior to commencing the therapy program participants were most concerned about therapy time (avoiding programs that were too intensive), and the amount of recovery they would make. Following the therapy program, participants were more concerned with the mode of therapy and preferred traditional therapy programs over programs using the Wii Fit.

**Conclusions:**

The usefulness of the Wii Fit as a therapy tool with hospitalised older people is limited not only by the small proportion of older people who are able to use it, but by older people's preferences for traditional approaches to therapy. Mainstream media portrayals of the popularity of the Wii Fit with older people may not reflect the true acceptability in the older hospitalised population.

## Background

Health and aged care services are exploring new ways of delivering therapy programs in the context of an aging population, increased demand for therapy services and scarce resources [1]. The use of interactive video games as a therapy tool for addressing both physical and cognitive function is a growing trend in the health and aged care sector [2]. There are thought to be several benefits evident in using video games in therapy. Firstly, while traditional therapy programs may suffer from poor compliance rates, video game activities are designed to be fun and motivating and therefore may increase the amount of time the user spends in therapeutic activity [3]. Secondly, the activities are graded, structured and provide detailed feedback on performance thereby incorporating factors thought to be important in learning new skills [4]. Thirdly, the games are accessible and affordable and can be used in a wide range of environments including the home, clinic or residential care setting. In addition, games may promote social interaction; this feature may be useful in residential care settings or may be valuable in providing shared interests between generations (for example older people playing the games with their grandchildren).

The Nintendo Wii video games console has been particularly popular and its evolving use in acute hospitals, residential care facilities and rehabilitation programs has been widely reported in the media [5-7]. A recent audit revealed that 61% of metropolitan stroke rehabilitation hospitals in Australia had purchased a Nintendo Wii console [8]. The Wii Fit program has been marketed as a health and fitness tool and was recently the first interactive video game to be endorsed by the United Kingdom's National Health Service Change4 Life program [9]. Wii Fit activities are designed to improve balance, aerobic capacity and strength and are displayed on a television screen as the user interacts with the program using a wireless remote control and balance board. Use of the Wii Fit program in health or aged care settings is generally limited to individuals that are able to step on and off the balance board, hold the remote control, have sufficient vision to see the game displayed on a screen, understand the concept of the game and comprehend the instructions. Activities can be adapted by therapists to suit individuals (for example, sitting on the balance board rather than standing [10]).

While there has been a widespread introduction of the Nintendo Wii video games console and Wii Fit program there have been few clinical trials to date of it's effectiveness as a therapy tool when used with older people [11-13]. Furthermore despite the marketing of the program towards people of all ages and prevalence of the program in hospital settings, there is little rigorous information about how acceptable the program is to hospitalised older people. A controlled study, which examined the acceptability of the Wii Fit as part of a community falls prevention program [14], reported that all of the participants in the Wii Fit intervention group (n = 15) found it to be enjoyable and acceptable and that 61% reported that they would prefer to continue with Wii Fit based therapy rather than attend a falls exercise group. However, it must be noted that study recruitment was achieved via a press release. Therefore, the group of participants recruited is unlikely to reflect the preferences of a broader group of older people.

We decided to measure acceptability of the Wii Fit program using a Discrete Choice Experiment (DCE); DCEs are increasingly used in health care as a rigorous technique to determine patient preferences for health care programs [15]. DCEs are based on two fundamental assumptions, (1) that health care interventions or services can be described by their characteristics (or attributes), and (2) that the consumer's valuation of the intervention or service is based on the levels of these attributes [16]. Attributes of a service (such as waiting time, location and staff providing the service) have been shown to be important to patients in addition to health care outcome [17,18]. DCEs are typically presented in a survey or questionnaire format in which the respondent is posed with repeated hypothetical choices between two different health care interventions (consisting of varying levels of attributes) in which they must identify the intervention that they prefer. Analysis enables the calculation of the levels and attributes that are most valued by respondents [16]. Advantages of DCEs over more traditional forms of determining patient preference are: they may be more likely to simulate real life decision making processes and they are not susceptible to the gratitude bias often seen in patient satisfaction surveys [19,20].

The aim of this study was to determine the acceptability of the Nintendo Wii Fit as a therapy tool for older people using a DCE before and after exposure to the intervention on a geriatric rehabilitation unit. It was hypothesised that participants would regard the Wii Fit intervention more positively after exposure to the program.

## Methods

This study was approved by the Southern Adelaide Clinical Human Research Ethics Committee (Study ID: 366/09). The study was nested within a pilot randomised controlled trial comparing the safety and effectiveness of the Wii Fit (when used in physiotherapy sessions) compared with a conventional physiotherapy approach. This paper reports on the use of the DCE with only those participants allocated to using the Wii Fit.

### Establishing the attributes and levels of the DCE

As this DCE was designed specifically to address the acceptability of the Nintendo Wii Fit as a therapy tool (a policy question), the attributes and levels of the DCE were determined by the researchers in collaboration with experienced geriatricians. Candidate attributes and levels of a hospital based physiotherapy program that were realistic and capable of being traded were described following a pilot study with older people on the unit. The final chosen attributes and levels are presented in Table 1.

**Table 1:** Attributes and levels used in the discrete choice experiment

*Attributes*	*Levels*
Mode of therapy	Conventional therapy
	Therapy using the Nintendo Wii
Difficulty of therapy	Easy - 30 minutes of light activity
	Challenging - 1 hour moderate activity
Cost	No cost
	$25 per week
	$50 per week
Amount of recovery made	70%
	80%
	90%

### Producing scenarios

We used a fractional factorial design and the techniques described in Street and Burgess [21] resulting in 12 binary choice sets which were 100% efficient for estimating the main effects. The 12 binary choice sets were divided into two versions of the questionnaire with six choice sets in each version to reduce the cognitive burden upon participants. An example of a choice set is presented in Table 2.

**Table 2:** An example of a choice set from the discrete choice experiment

*Program 1*	*Program 2*
Therapy using Nintendo Wii	Traditional therapy
Easy - 30 minutes of light activity	Challenging - 1 hours moderate activity
$25 per week	$50 per week
80% recovery	70% recovery

### Administering the questionnaire

Participants were recruited from a geriatric rehabilitation unit at the Repatriation General Hospital, a 300 bed acute care hospital in metropolitan Adelaide, South Australia. Inclusion criteria were: aged ≥ 65 years, Mini Mental State Examination (MMSE) [22] score of ≥ 21/30 (regarded as mild or absence of cognitive impairment [23]), medically fit to participate (as determined by the treating medical team), weight of < 150 kg, able to perform sit-to-stand transfers without physical assistance, ambulating independently prior to admission and adequate vision (determined as 6/18 on a Snellen Chart).

Patients admitted between May and December 2010 were approached and those who consented to participate completed the first DCE via an interview administered questionnaire within 24 hours of admission to the unit. The six choice sets involving therapy programs were presented to the participant sequentially and the participants were asked to choose which therapy program they preferred. Participants were shown pictures of the two alternative therapy approaches (older people using the Wii Fit or participating in more traditional exercises in a therapy gym) in order to assist them in understanding the alternatives. Both therapy approaches were described as being of the same intensity and both located on the geriatric rehabilitation unit. The second DCE was administered one month after discharge giving the person time to reflect on their hospital stay and experience of therapy.

Study participants used the Wii Fit as prescribed and supervised by the treating physiotherapist for 25 minutes per day, five days per week for the duration of their stay. Participants were not involved in any additional ward based physiotherapy activities. Wii Fit activities were prescribed based on the participant's abilities and needs. Activities included balance games in which the user has to shift their weight laterally on the balance board (such as attempting to 'balance on an iceberg'), strength tasks (such as sustained squats or single leg extensions) and light aerobic tasks such as walking on the spot. All activities were performed while standing and safety equipment (for example a sturdy chair or the person's walking aid) and rest breaks were used as required. The treating physiotherapist familiarised the participants with the program, selected all activities, operated the Wii Fit program (for example turning on and navigating through the menus) and provided close standby assistance. The games were played with only the physiotherapist present and presented to the participant as a therapeutic task rather than a social activity.

### Data Analysis

The data were analysed using conditional (fixed-effects) logistic regression in STATA. Effects coding was used [16] and the base cases (conventional therapy, light activity, no cost and 70% recovery) excluded from the analysis. Statistically significant coefficients demonstrate the importance of that attribute in influencing preferences and determining overall utility. Coefficients with a positive sign show that respondents valued that particular attribute, whereas coefficients with a negative sign show that respondents were averse to that attribute.

## Results

### Characteristics of respondents

**Figure 1:**
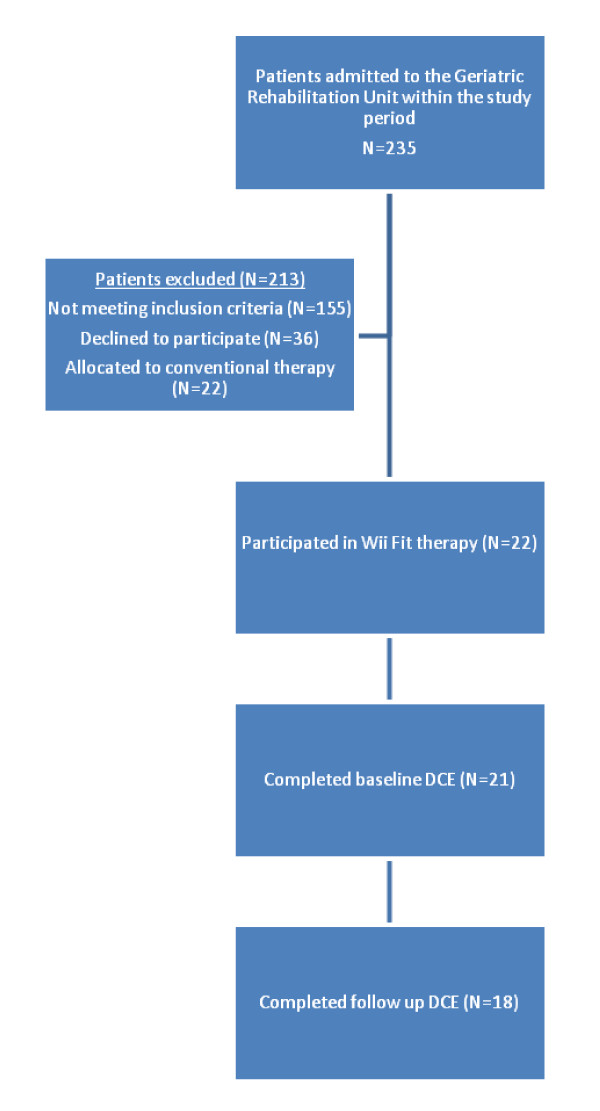
**Recruitment of study participants**.

Participants (N = 21) had a mean age of 85.4 years (SD 4.7) and the majority were female (86%). Participants' mean length of stay on the geriatric rehabilitation unit was 12 days. Participants were relatively high functioning; all were able to perform sit-to-stand transfers without physical assistance and the mean score on the MMSE was 26 (SD 2). Participants participated in an average of six (SD 2.4) sessions using the Wii Fit with high levels of adherence (90%). Three participants did not complete the DCE following intervention (one participant withdrew from the study and two participants experienced significant cognitive decline within the study period and were no longer able to adequately understand the questions) therefore the data from 18 participants was included in the follow up analysis. Details of recruitment are presented in Figure 1.

### Discrete choice experiments model estimation

Results of the DCE analysis are presented in Table 3.

**Table 3:** Discrete choice experiment model results for respondents

	*Baseline (n = 21)*					*After intervention (n = 18)*				
**Characteristic**	**Coefficient**	**P**	**95% CI**			**Coefficient**	**p**	**95% CI**		

Wii Fit	-0.2019	0.200	-0.5107	to	0.1068	-0.4949	0.001*	-0.7828	to	-0.2070

Challenging	-1.1200	0.000*	-1.5126	to	-0.8880	-0.5709	0.000*	-0.8589	to	-0.2829

Cost $25	-0.3097	0.154	-0.7357	to	0.1162	-0.0198	0.925	-0.4293	to	0.3898

Cost $50	0.2162	0.333	-0.2215	to	0.6539	-0.4266	0.047*	-0.8467	to	-0.0066

Recovery 80%	0.0178	0.940	-0.4450	to	0.4805	-0.1020	0.617	-0.5024	to	0.2984

Recovery 90%	0.8093	0.000*	0.3193	to	1.2272	0.4985	0.021*	0.0759	to	0.9211

* Significant at 5% or better level

At baseline it can be seen that the mode of therapy offered (Wii Fit or conventional) did not significantly influence the respondent's choice of therapy program. The attribute with the highest negative value was challenging therapy (1 hour of moderate activity) reflecting patient's aversion to longer and more demanding therapy sessions. Patients also significantly favoured therapy programs that would result in the maximum amount of recovery (90%).

The results from the follow-up DCE show a change in participants' preferences. It appears that the mode of therapy offered became significantly more important to participants, and respondents preferred therapy programs that did not use the Wii Fit. The cost of the therapy program was also somewhat more important with participants indicating a reluctance to pay $50 per week for the therapy program and remaining averse to challenging therapy programs of one hour duration and attracted to programs that would result in maximum recovery. The interviewer administering the DCEs reported that while respondents were completing the follow-up DCE they often commented that they perceived that conventional therapy was more effective than therapy using the Wii Fit.

## Discussion

Our key finding challenges the widespread uptake of the Wii Fit by rehabilitation units. Our hypothesis that participants would regard the program more positively after exposure to the intervention was not supported by our data. While initially the participants reported no strong preference for the way in which their therapy was delivered (conventional therapy or computer based), after experience using the Nintendo Wii Fit, they reported an aversion to this type of therapy and a preference for conventional therapy approaches often citing they felt conventional therapy to be more effective.

In contrast to previous portrayals of the Nintendo Wii Fit as a popular new therapy approach with older people [5-7,13,14], our findings suggest that mainstream media portrayals of the popularity of the Wii Fit with older people may not reflect the true acceptability of this technology in the older hospitalised population. Furthermore, our participants are likely to be more open minded than average about therapy options as they went through a consent process and could have opted out.

This study did not aim to explore the reasons as to why participants preferred a conventional approach to therapy and this is an area for further research. Exploring why participants felt that conventional therapy was more effective would also be valuable, as the use of technology in rehabilitation is likely to increase. It is possible that the participants preferred more interaction with therapy staff or felt that the technology was too complex. It is also possible that the characteristics of the Wii Fit hardware and software were disliked by the participants, for example, perhaps they found the games too childlike or too challenging. Interactive gaming programs that are specifically designed for older people in rehabilitation may be more acceptable; this has been suggested by previous studies which reported high levels of participant satisfaction with specialised rehabilitation games [3,24,25].

It is possible that this population of older people may have valued the Wii Fit more highly if research demonstrated that use of the program resulted in more positive outcomes than conventional therapy. However, there is a body of literature suggesting that patients' preferences for health care services are influenced by a 'veil of experience' in which they tend to prefer the services with which they are most familiar [26-28]. While the participants may not have received hospital based physiotherapy previously, it is reasonable to assume that (in contrast to the Wii fit program) most of the participants would be familiar with the sorts of activities and exercises provided in this context. This bias against new innovations and technologies may be particularly strong in older people, and may have been apparent in our participant group. It is also possible that participants may have regarded the Wii Fit more highly if they participated in a greater number of therapy sessions. Participants took part in an average of six sessions of 25 minutes using the Wii Fit. It is possible, therefore, that they may have required more time and more sessions to become familiar with and properly engage with this approach to therapy. Increased familiarity with the program may have also enabled participants to choose their favourite activities rather than activities being selected for them by the physiotherapist which may have increased their level of enjoyment. Furthermore, the inclusion of participants that may have had mild cognitive impairment may have resulted in these participants finding the games too complex and resulting in reduced satisfaction.

One of the limitations of this study is the small sample size, and studies with a larger number of participants are required to confirm the results of this study. As this study took place in a unit where most respondents were aged over 80 years, the clinical usefulness and acceptability of the tool may be greater in younger rehabilitation clients. The context of the use of the Wii Fit must also be taken into account; this study examined it's acceptability as a therapy tool used by physiotherapists as part of a hospital therapy program therefore older people's perceptions of the Wii Fit may be different when used for example alone in a home setting.

One of the advantages of using a DCE to determine the acceptability of a therapy approach is that enables us to determine the relative importance of some attributes in comparison to others. In hindsight, it would have been useful to ask the participants simply whether they felt that the use of this sort of interactive gaming program was a useful therapy approach and compare this to the results of the DCE.

The advantages of computer games in rehabilitation settings include their relative inexpense and potential to be self motivating and it is likely that their role in rehabilitation will increase. Our small study however suggests that issues around uptake and acceptability still need to be addressed in clinical populations.

## Conclusions

Although new technologies may offer advantages over traditional approaches, in order to provide services that are acceptable, there needs to be an awareness of patient preferences. This study showed that after trying the Wii Fit on a geriatric rehabilitation unit, participants indicated that they preferred traditional therapy approaches. The results suggest that new technologies in rehabilitation may only be popular with a small group of patients at present and therefore implementation decisions should take into account their cost relative to their likely uptake rates. Older people may be more accepting of new technologies if their effectiveness was demonstrated in clinical trials although this may not outweigh people's preferences for more traditional approaches, or approaches with which they are familiar.

## Competing interests

The authors declare that they have no competing interests.

## Authors' contributions

KL participated in the design of the study, collected and analysed the data and drafted the manuscript. JR was involved in the design of the study, interpretation of the analysis and drafting the manuscript. SG was involved in the design of the study and drafting the manuscript. LB designed the DCE and was involved in drafting the manuscript. MC was the chief investigator and was involved in the design of the study, interpretation of analysis and drafting the manuscript. All authors read and approved the final manuscript.

## Pre-publication history

The pre-publication history for this paper can be accessed here:

http://www.biomedcentral.com/1471-2318/11/64/prepub
